# Pituitary Adenomas and Invasiveness from Anatomo-Surgical, Radiological, and Histological Perspectives: A Systematic Literature Review

**DOI:** 10.3390/cancers11121936

**Published:** 2019-12-04

**Authors:** Simona Serioli, Francesco Doglietto, Alessandro Fiorindi, Antonio Biroli, Davide Mattavelli, Barbara Buffoli, Marco Ferrari, Claudio Cornali, Luigi Rodella, Roberto Maroldi, Roberto Gasparotti, Piero Nicolai, Marco Maria Fontanella, Pietro Luigi Poliani

**Affiliations:** 1 Neurosurgery, Department of Surgical Specialties, Radiological Sciences and Public Health, University of Brescia, 25123 Brescia, Italy; s.serioli002@studenti.unibs.it (S.S.); alessandro.fiorindi@gmail.com (A.F.); antoniobiroli@yahoo.com (A.B.); claudio.cornali@unibs.it (C.C.); marco.fontanella@unibs.it (M.M.F.); 2 Neurosurgery, Spedali Civili Hospital, 25123 Brescia, Italy; 3 Otorhinolaryngology–Head and Neck Surgery, Department of Surgical Specialties, Radiological Sciences and Public Health, University of Brescia, 25123 Brescia, Italy; davide.mattavelli@unibs.it (D.M.); 1990marcoferrari@gmail.com (M.F.); piero.nicolai@unibs.it (P.N.); 4 Section of Anatomy and Physiopathology, Department of Clinical and Experimental Sciences, University of Brescia, 25123 Brescia, Italy; barbara.buffoli@unibs.it (B.B.); luigi.rodella@unibs.it (L.R.); 5 Radiology, Department of Surgical Specialties, Radiological Sciences and Public Health, University of Brescia, 25123 Brescia, Italy; roberto.maroldi@unibs.it; 6 Neuroradiology, Department of Surgical Specialties, Radiological Sciences and Public Health, University of Brescia, 25123 Brescia, Italy; roberto.gasparotti@unibs.it; 7 Section of Pathology, Department of Molecular and Translational Medicine, University of Brescia, 25123 Brescia, Italy

**Keywords:** anatomy, cavernous sinus, classification, diagnosis, histology, invasiveness, pituitary adenoma, PitNET, radiology, surgery

## Abstract

Invasiveness in pituitary adenomas has been defined and investigated from multiple perspectives, with varying results when its predictive value is considered. A systematic literature review, following PRISMA guidelines, was performed, searching PubMed and Scopus databases with terms that included molecular markers, histological, radiological, anatomical and surgical data on invasiveness of pituitary adenomas. The results showed that differing views are still present for anatomical aspects of the sellar region that are relevant to the concept of invasiveness; radiological and histological diagnoses are still limited, but might improve in the future, especially if they are related to surgical findings, which have become more accurate thanks to the introduction of the endoscope. The aim is to achieve a correct distinction between truly invasive pituitary adenomas from those that, in contrast, present with extension in the parasellar area through natural pathways. At present, diagnosis of invasiveness should be based on a comprehensive analysis of radiological, intra-operative and histological findings.

## 1. Introduction

**Figure 1:**
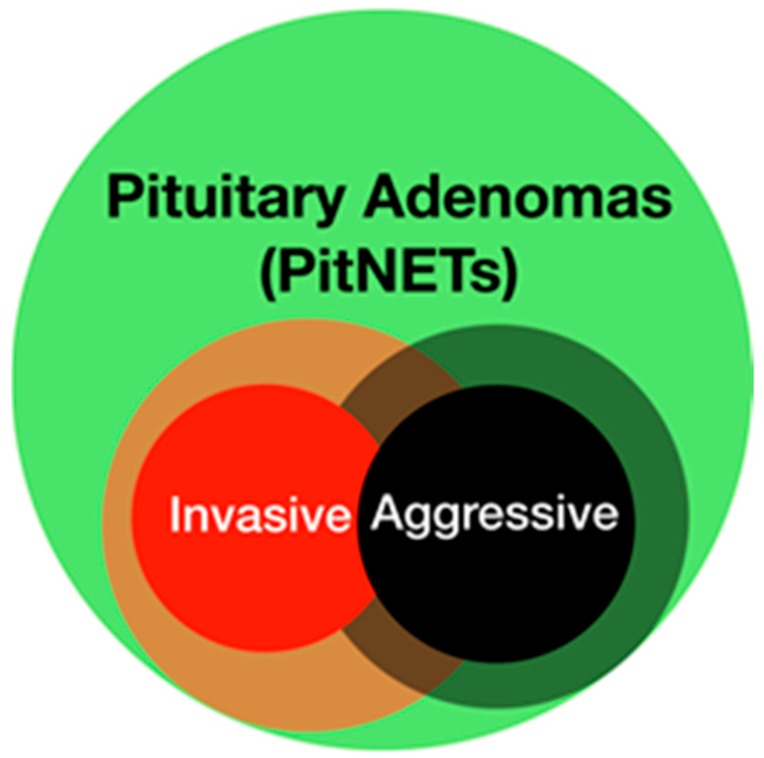
Theoretical representation of pituitary adenomas (PAs). Invasive and aggressive PAs represent two subgroups of the main population, which overlap. The blurred region underlines that both definitions can vary according to different authors and points of view. In clinical practice, an aggressive pituitary adenoma with a high proliferative index is usually invasive. A pituitary adenoma that extends in the cavernous sinus is frequently considered invasive from a radiological point of view, but might not be from surgical or histological points of view; on the other hand, in theory, even a small, intrasellar pituitary adenoma might be invasive.

Pituitary adenomas (PAs), also defined as pituitary neuroendocrine tumors (PitNETs) as explained in the following sections, are generally benign and slow growing tumors that can cause a “mass effect” on nearby anatomic structures, which are generally displaced or remodeled over time (e.g., sellar enlargement). The main features of some PAs include: 1) more aggressive biologic behavior, which typically corresponds to a proliferative index that is higher than usual; 2) an invasive behavior, so that the PAs extend in the extrasellar region and/or histologically invade the surrounding dural, periosteal, or mucosal tissues. Aggressiveness and invasiveness are sometimes used as synonyms, as the two features are frequently present in the same tumor, but they should not be confused as they describe two distinct characteristics of PAs (Figure 1) [1,2,3,4,5,6,7]. 

Furthermore, while aggressiveness is generally defined by the clinical behavior of the lesion, invasiveness can be described in different ways, according to the point of view. The aim of this study is to provide a systematic review of the concept of invasiveness from anatomo-surgical, radiological, and histological perspectives.

## 2. Literature Review

A total of 5555 papers were identified after removal of duplicates. After title and abstract analysis, 1496 articles were identified for full-text analysis. Eligibility was ascertained for 590 articles; 221 studies were included in the systematic review (see Section 3 for further details). The results were subdivided according to three main perspectives: anatomo-surgical, radiological, and histological. The following sections present a critical discussion of the most significant results.

### 2.1. Epidemiology

The reported frequency of invasive PAs varies considerably, from 2–3% [8] to 21% [9,10]. Predilected sex remains matter of debate, although they seem to be larger and more frequent in men [11,12,13,14]; conflicting results are reported for age incidence [15], including those in childhood and adolescence [16,17,18,19,20].

Regarding hormone secretion, invasive tumors are more frequently non-functioning, silent corticotroph and silent subtype-three PAs (now defined plurihormonal PIT1-positive adenomas, according to the most recent WHO classification [21]), or PRL-secreting and GH-secreting [22,23,24,25,26,27,28,29,30]. Sphenoid invasion seems to be related to the size of the PA [31] and male gender [12]. However, clivus encroachment seems to be prevalent in women [32].

### 2.2. Anatomo-Surgical Considerations on Invasiveness

#### 2.2.1. Anatomy of the Sellar and Parasellar Region

A precise definition of the anatomy, both macroscopic and microscopic (i.e., including the collagen layers), of the sellar and parasellar regions is essential to distinguish between invasive PAs and non-invasive tumors that might extend in the parasellar region through natural pathways (see graphical abstract).

The description and definition of the collagen layers that define the sellar and parasellar region have been addressed by different authors; significantly different data have been reported. 

**Figure 2:**
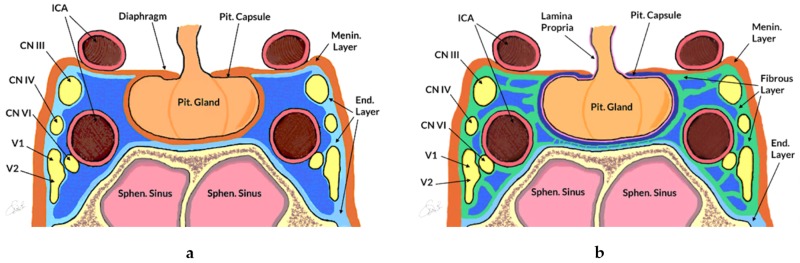
Two differing models of the sellar and parasellar anatomy. (**a**) Schematic drawing, according to Yasuda et al. [38]. The pituitary capsule (black line) wraps the hypophysis. The medial wall of the cavernous sinus is formed by a single meningeal layer (in orange), which is a continuation of the diaphragm sellae, whereas the lateral and superior wall have a double coat: the inner one is defined as the endosteal layer (in light blue), which covers the sellar floor and cranial nerves that are at the level of the lateral wall of the cavernous sinus (i.e., III, IV, V1-2, while VI and intracavernous segment of ICA are not involved); the outer one is the meningeal layer. (**b**) Schematic drawing, according to Songtao et al. [39]. The pituitary gland is covered by connective tissue defined lamina propria (in pink). The medial wall is formed by two different layers: the pituitary capsule (in blue) and the fibrous layer (in green). The supero-lateral coat consists of the meningeal (in orange) and the fibrous (in green) layers. The fibrous layer surrounds all the arterial and nervous structures of the parasellar compartment. In both anatomical models, the endosteal layer (in light blue) coats the sellar floor. CN—cranial nerve; End. Layer—endosteal layer; ICA—internal carotid artery; Menin. Layer—meningeal layer; Pit. Capsule—pituitary capsule; Pit. Gland—Pituitary Gland; Sphen. Sinus—sphenoid sinus.

According to some authors, the hypophysis is covered by a thin layer, called the pituitary capsule (Figure 2a) [33,34,35,36,37,38]. It is easily identifiable and separable from the medial wall of the cavernous sinus (MWCS) [33,36] and is formed by dense fibrous tissue with thickness ranging from 10 to 60 μm [33]. Songtao et al. named the layer closely adhering to the gland the “lamina propria”, which is formed by connective septations penetrating the gland and with constant thickness (22.48 ± 5.88 μm); they referred to the medial wall layer with the term pituitary capsule (Figure 2b) [39]. Destrieux et al. [40] used the term “dural bag” to describe the only limit between the pituitary gland and the cavernous sinuses (i.e., the meningeal layer according to Yasuda et al. [38] —Figure 2a). Immunohistochemical analysis has shown that various types of collagen contribute to this fibrous casing, including collagen types I [33,35,39], II [33], III [33,39], V [33,39] and IV, the latter being the most frequent [33,35,39,41].

The medial wall of the cavernous sinus (MWCS) represents the border between the sella and cavernous sinus (Figure 2). Variations in definitions and morphology have also been reported for this structure [42]. The MWCS superiorly is folded to form the diaphragma sellae [37,38,39,43], whereas inferiorly it is formed by the periosteal dura of the sella turcica [40,44]. The mean thicknesses vary according to different authors [33,36,38,45,46], ranging from 10 to 387 μm; it is always less than the thickness of the superior and lateral cavernous sinus (CS) walls, and tends to decrease from anterior to posterior [36] and from inferior to superior [46]. Through the use of diaphanoscopy, Knappe et al. studied the MWCS on 14 formalin-fixed sellar emiblocks. The thinning was evident at three specific points: laterally to the sellar boundary (93%), below the horizontal segment of the ICA (71%) and antero-inferiorly to the anterior knee of the carotid siphon (64%) [47]. 

Some studies have also reported defects in the cavernous sinus walls, which could explain PAs with extensions beyond the sellar region [33,36,45,48]. Yokoyama et al. described small defects in the medial wall (capsule of the pituitary gland) in 3 of 30 sections [45]. Kawase et al. found three weak points of CS: at the level of venous plexus of SOF, at the medial wall of pituitary gland, and at the cisternal segment of the intracavernous portion of IIIrd and Vth cranial nerves [48]. According to some authors, macro-PAs may indeed show a preferential growth pathway extending into the parapeduncular space through the oculomotor triangle [49,50,51,52]. The dural layer of the cistern of the oculomotor nerve could be thin or even lacking, representing a possible point of diffusion exploitable by PAs [49].

Although the existing evidence has described some conflicting results, the described reduction in thickness and the defective sites of MWCS might explain the parasellar growth pattern of PAs that would not have to be biologically invasive, even if they might present with radiological signs of invasion: the extrasellar extension of this subgroup of PAs might be supported by the hypothesis of a path of least resistance, which might be followed even by a non-invasive PA [33,45,48].

Immunohistochemical studies of MWCS showed conflicting results. A positivity for collagen types I [33,39,41], II [33,39], III [41] and IV [35] has been reported. A true invasion of the cavernous sinus by PAs might be based on the expression of collagenase, in particular MMP-9 and -2, facilitating the invasion and destruction of the collagen layers [17,53,54,55,56,57]. However, further studies are needed to better investigate this aspect.

#### 2.2.2. PAs and Surgical Invasiveness 

Surgical observations have the fundamental role of providing data for diagnosis of extension or invasion of PAs [7,58,59,60,61].

From a surgical point of view two anatomical corridors have been identified relative to the ICA: the medial and lateral ones [58,62,63,64].

The medial corridor may have a tetrahedron or hexahedron shape, and is delimited laterally by the C portion of ICA (C4 and C5 segment), medially by the MWCS, posteriorly by the petroclival ligaments and posterior clinoid process; the lateral one is located between the anterior genu and horizontal segments of ICA, medially, and LWCS, laterally [58,65,66]. 

Knosp et al. [67] divided the cavernous space into four venous compartments with regards to the ICA: the medial, superior, lateral and inferior compartments. If there was compression of three or more venous compartments or the lateral venous compartment, surgical diagnosis of invasion was made [67]. Connor et al. [68] reported that extension in the inferolateral or lateral venous compartments was related with incomplete surgical resection. Fernandez-Miranda et al. [69] proposed a surgical anatomy classification of the CS, distinguishing between four different compartments (superior, posterior, inferior, and lateral). In their work, the most commonly invaded compartment was the superior (65 patients, four with bilateral invasion), followed by the posterior (60 patients, six with bilateral involvement), inferior (34 patients, seven exhibiting bilateral involvement), and lateral (23 patients, one with bilateral invasion). In cases with multiple compartment invasion, the most common pattern was the superior/posterior (*n* = 32) followed by the inferior/superior/posterior/lateral (*n* = 17), inferior/superior/posterior (*n* = 14), and superior/lateral (*n* = 11). It was found that no patients had growth into the inferior/posterior/lateral compartments without invasion of the superior compartment [69]. Trevisi et al. [70] proposed a four-quadrant classification derived from the clock method previously suggested by Moreau et al. [71,72] (see Radiology section for further details). A higher rate of GTR was seen, when one or two quadrants were invaded (respectively 86% and 70%) or when the SM (superomedial), SL (superolateral), and IM (inferomedial) quadrants were involved [70].

These data are recent and have become available thanks to the introduction of the endoscope in transsphenoidal surgery [73]: thanks to its panoramic view, surgeons are now able to directly visualize the MWCS, thus collecting more data to distinguish between PAs extending in the parasellar region through compression or extension, from those that are truly invasive. Furthermore, MWCS resection and surgical removal of soft PAs in the lateral compartment is possible in experienced hands [62,63,74,75,76,77]. As surgeons, it remains impossible to provide a diagnosis of microscopic invasion, at least with the available technology.

Furthermore, recent papers have underlined the possible advantages of intraoperative MRI [78,79,80,81,82,83], which can provide additional data especially in cases of truly invasive PAs, as the surgeon might not be able to visualize tumor that has grown behind invaded normal tissue. 

### 2.3. Radiology and PAs Invasiveness

The radiological study represents the first essential step for evaluation of PA invasiveness. Tumor size plays a key role: the larger the lesion, the greater the correlation with invasiveness [9,10,12,15,30,31]. The tumor may infiltrate many structures: the sellar floor, involving inferiorly the sphenoid sinus and nasopharynx; the cavernous sinus laterally, with an incidence ranging from 10% [67,71] to 21% [10]; superiorly, the tumor frequently extends in the suprasellar region, rarely invading the arachnoid [84,85,86]; anteriorly, it might extend in the ethmoid and orbital region; posteriorly, in the clivus (8.2%) [32] and, rarely, in the posterior fossa [11,87]. Inferior extension seems to be more typical in older patients, while patients with cavernous sinus invasion are usually younger [15]. Sphenoid invasion seems to be related to the size of the PA [31] and to male gender [12].

#### 2.3.1. Radiological Criteria and Classifications

**Figure 3:**
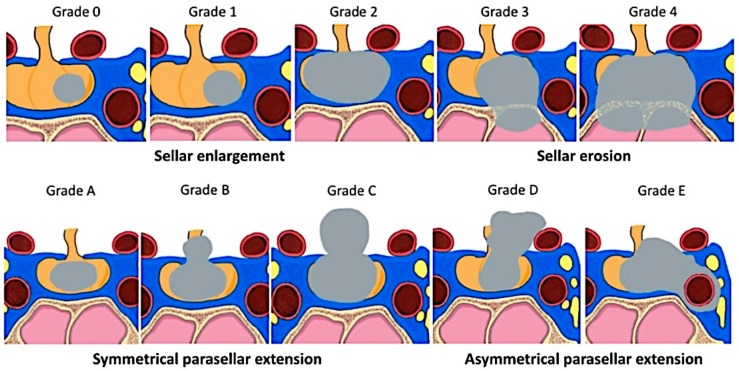
Hardy’s classification of pituitary adenomas [88] and Wilson’s modification [90]. The founder of microsurgical transsphenoidal surgery, Jules Hardy [88,89], suggested a classification that included distinction between sellar enlargement and sellar erosion, with grades III and IV defined as “likely invasive” [3,91]. His classification of parasellar PAs was partially modified by Wilson to distinguish between different extrasellar extensions, including extension in the cavernous sinus (grade E) [90]. Recently, a simplified, dichotomized version of Hardy’s classification has been suggested and validated [92].

Several radiological classifications have been proposed to predict invasiveness in PAs since the introduction that suggested by Jules Hardy, who distinguished between sellar enlargement and invasion [88,89] (Figure 3). This classification was soon partially modified to include a grade that describes the extension of the adenoma within the cavernous sinus (Figure 3). 

**Figure 4:**
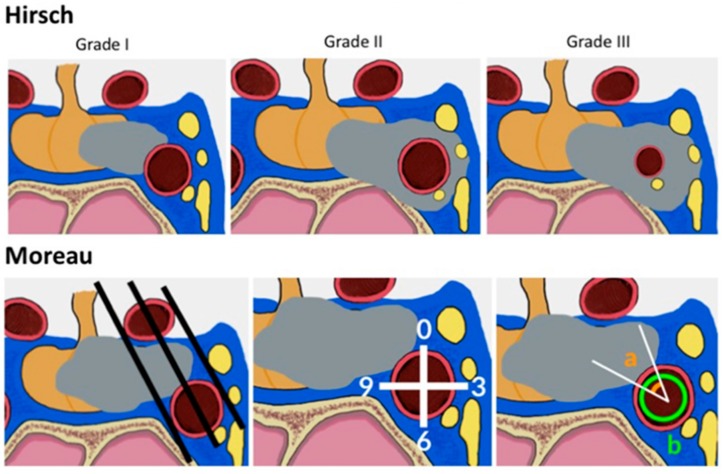
Summary of radiological criteria of cavernous sinus (CS) extension of sellar tumors. (a) Hirsch et al. [93] suggested three grades to define encasement of the parasellar ICA for tumors invading the CS: in grade 1, the tumor partially touches the ICA; in grade 2, the ICA is encircled without luminal narrowing, and in grade 3 the ICA diameter reduction is evident. This staging system is not usually used for PAs, but rather for all lesions found in the parasellar region [106,107,108]. Cavernous ICA stenosis is rarely found in PAs. (b) Moreau and coworkers [71,72] defined three criteria to investigate CS invasion, based on intercarotid lines, the subdivision of the cavernous ICA in quadrants, and analysis of the angle defined by the ICA surface in contact with the PA.

As the invasion of cavernous sinus is indeed a significant limiting factor for surgical resection, different criteria and classifications have been suggested to better predict this feature pre-operatively (Figure 3 and Figure 4).

The initial radiological criteria of CS invasion, which underlined the importance of cavernous sinus ICA (Figure 3) [71,93,94], have been further refined and include analysis of the pituitary gland (its interposition between the tumor and CS is a sign of no invasion), of the CS venous compartments, CS size, ICA, and CS lateral wall (CSLW) displacement [68,71,95,96,97,98,99,100,101]. All these criteria underline the difficulty of radiological diagnosis of CS invasion, which is considered certain only when total encasement of the ICA is present [11,67,71,87,94,95,97,101,102,103,104,105].

**Figure 5:**
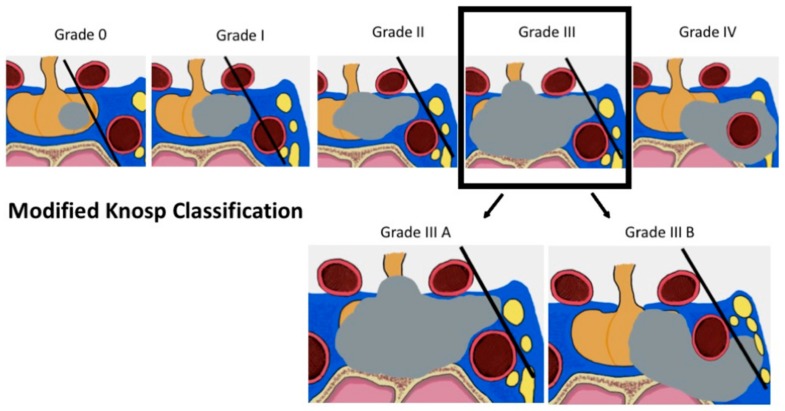
Knosp classification of CS extension of PAs. Initially described in 1993, Knosp grading of PAs extending in the cavernous sinus was modified in 2015 by the same group. Novel data, acquired in the endoscopic era, have shown that grade 3A PAs are less frequently invasive than grade 3B. This difference might be related to the different anatomy of the MWCS, as grade 3A tumors might displace it or extend in the cavernous sinus through defects (see also Graphical Abstract and text for further details).

The most widely used classification of CS involvement was suggested by Knosp in 1993 [67]. In the microsurgical era, Knosp et al. introduced a classification of the parasellar extension of PAs based on coronal resonance imaging [67]. Three lines (medial, median and lateral), which cross the internal carotid artery (ICA), determine the degree of invasion (Figure 4). Grade four corrisponds to complete encasement of the ICA by the tumor. In their study, parasellar extension was surgically checked [67]: all Grade 3 and 4 PAs and almost all Grade 2 PAs were intraoperatively classified as invasive. In 2015, the group led by Knosp suggested a subdivision of grade 3 into 3A and 3B (Figure 5): in Grade 3A, the tumor extends laterally in the superior compartment of the cavernous sinus, whereas in Grade 3B the lesion extends laterally, but in the inferior compartment [60]. The same group has shown that grade 3A PAs have a lower rate of invasive growth than adenomas extending into the inferior cavernous sinus compartment or encasing the carotid artery (i.e., grade 3B and 4) [109]. This finding is clinically and biologically relevant, and is most probably the consequence of better surgical visualization provided by an endoscope: the improved possibility of visually checking the medial wall of the cavernous sinus intraoperatively, behind the cavernous ICA loop, has shown that the medial wall can be frequently displaced and not invaded in grade 3A PAs; in other cases, it can be spontaneuosly dehiscent and provide a natural corridor for extension of PAs in the cavernous sinus [62].

Other radiological classifications, which include features of the previous ones, have been suggested to attempt an in detail analysis of PA extensions [110], or provide a radiological pathology-specific grading [83,111,112,113,114].

Preoperative radiological diagnosis of CSI plays a crucial role in establishing prognosis and the therapeutic plan. Although we cannot exclude that even an adenoma with Knosp grade ≤ 2 may be invasive [115], pituitary tumors with Knosp grades 3B–4 are characterised by subtotal surgical resection, low biochemical remission rate [10,11,111,116,117,118,119,120,121], need for complementary therapies, and a higher risk of recurrence [84]. However, radiology does not always allow discriminating between compression/extension and invasion of CS, and it might therefore overestimate CS invasion.

#### 2.3.2. Radiological Technical Advancements

Different technical aspects have been described to improve the radiological accuracy of a diagnosis of CS invasion. Three-tesla MR imaging is superior to standard 1.5T MR imaging, in particular for examining the medial wall of the cavernous sinus (MWCS) [122]. Cao et al. tried to better characterize the dural layers that form the MWCS through the use of proton-density-weighted imaging (PDWI), identifying defects of the MWCSs and distinguishing between compression and invasion of CS [123]. However, the MWCS is a very thin dural layer and it is difficult to establish its involvement by tumors through MRI only [68]. Other sequences have been suggested as well, including VIBE [124], CE SPACE (contrast enhanced sampling perfection with application-optimized contrasts using different flip-angle evolutions) [125], and T1-weighted three-dimensional (3D) fast spin echo (FSE) [126].

Recently, radiomics is playing an increasingly important role: its preoperative prediction of CS invasion based on contrast-enhanced T1 and T2-weighted magnetic resonance (MR) imaging might support clinical decision-making and predict outcomes [127]. Three-dimensional anisotropy contrast (3DAC) MR imaging [128], CE-MRA 3T (contrast-enhanced MR angiography) [129] or CE 3D FIESTA (contrast-enhanced 3D fast-imaging employing steady-state acquisition) [130] might be used to evaluate CS invasion and study the involvement of its cranial nerves.

### 2.4. Histological Evidence of Invasiveness

Dural invasion by PAs can be sometimes histologically documented, as previously reported in different studies [9,131]. Infiltration of the adjacent dura is recognized when single tumor cells or small aggregates are embedded within the dural layers [9,132]. Generally, this finding is associated with a high proliferative index [133,134] and increasing tumor size [59,132,133], although it has been reported in microadenomas with a low proliferative index, suggesting that invasive behavior may be related to the intrinsic biological properties of PAs rather than the rate of tumor growth [9]. In their work, Selman et al. [131] estimated that invasiveness defined by histological analysis is a more frequent finding (51 of 60 cases; 85%) than its evaluation at surgery (performed with the microscope in 1986). Apparently contrasting data have been reported by Scheithauer et al., who reported an invasion rate of 35% in 365 PAs [9]. The discrepancy between different studies reflects the problematic assessment of invasion based only on histological features and in the absence of valid biomarkers. Interestingly, stratifying the data based on the different types of PAs, invasion was 50% higher for GH (50%), PRL (52%), silent ACTH (82%) and TSH (75%) PAs, along with plurihormonal PAs (50%) and in Nelson’s syndrome (64%). As expected, invasion was found at a higher rate in pituitary macroadenomas extending outside the sella [9]. Of note, according to Meji and colleagues [132], patients who underwent trans-sphenoidal surgery for the first time showed dural invasion in 54.2% of null-cell adenomas, while this percentage decreased to 30-35% of patients in other PAs; in patients who underwent repeated pituitary surgery, the incidence of dural invasion was increased up to 90.9% for gonadotroph, 75% for PRL, and 73.3% for ACTH secreting PAs. Lonser et al. [59] reported histological dural invasion in 34% patients with CD: in 60% of cases, the invasion occurred at the level of the medial wall of the cavernous sinus, and in the remaining 40% it was found in other regions of the CS. Nishioka et al. [77] reported invasion in 71% of 150 acromegalic patients from a histological perspective and in 37% from a radiological point of view.

Since dural invasion can be studied radiologically, in spite of limitations, intraoperatively and in particular microscopically, different considerations on which strategies to utilize have been reported. According to Scheithauer et al., surgical or neuroradiological findings are endowed with a greater decisional value compared to the pure histological evidence of invasion [9]. It has to be considered that histological assessment of invasion is strictly dependent on both technical issues and tissue sample representativeness, which might frequently not allow its adequate evaluation.

### 2.5. Pathological and Molecular Considerations

Assessment of proliferative activity and histological and/or clinical evidence of invasion has been recommended by the recent WHO classification of pituitary tumors [21] for the identification of putative aggressive PAs. However, evaluation of tumor aggressiveness and invasiveness is still a debated issue. In addition, the previous definition of “atypical adenomas” has been removed from the WHO classification, since the effort to classify a subgroup of putative aggressive PAs based on the detection of mitoses and expression of Ki-67 and/or p53 has proven to lack reproducibility, without accurately predicting recurrences [135]. To this end, the international pituitary pathology club [136] has recently suggested to introduce the term pituitary neuroendocrine tumour (PitNET), according to terminology that is widely accepted in other neuroendocrine tumors (NETs) with the intent to obtain an adequate, prognostic grading system. Moreover, the simplistic distinction between PA and pituitary carcinoma, based only on metastatic spread, does not take into account the phenotypical, immunophenotypical, and molecular features of the lesion that reflects the different cellular biology and better correlates with clinical behavior. To overcome these limits, a grading system based on the combination of clinical and histopathological features, which correlates with high accuracy with the clinical outcome in term of recurrence rate and disease persistence, has been recently proposed by Trouillas et al. [137]. This system has been validated in independent reports [61,138] and included as integrated diagnostic information within the algorithm proposed for a standardized diagnostic approach to PitNETs by the European Pituitary Pathology Group (EPPG) [139]. The proposed grading system is basically founded on three major tumor features related to tumor size, proliferation, and invasiveness, while expression of p53, previously adopted as a potential marker of PitNET aggressiveness, has been confined to a minor role, since the low frequency of p53 detection by immunohistochemistry indicates that it is inadequate as a routine marker of invasiveness. Accordingly, our review of the literature confirmed that expression of p53 is a unclear issue, since only 40% of reports ascribe a predictive role to p53 expression with regards to invasive behavior, with the remaining reports highlighting uncertain and discordant results [140,141,142,143]. It has to be noted that p53-positive PAs have been reported to correlate with a significantly higher proliferation index (assayed by Ki-67/Mib1 immunohistochemistry) in invasive PAs, suggesting a major role of the proliferative features of these tumors [144]. In this regard, the proposed clinic-pathological grading system does not essentially differentiate from the previous indications of the 2004 WHO classification in that evaluation of the proliferation index is still founded on expression of Ki-67 and mitotic count (>3% with at least 2/10 mitoses/HPF). The real novelty is the integration of the evidence of invasiveness, defined as histological and/or radiological (MRI) signs of cavernous or sphenoid sinus invasion, within the grading criteria, according to the notion that invasion has been consistently associated with disease recurrence or persistence [137]. The authors also suggested that histological evidence of invasion of the dura, sphenoidal bone, or respiratory mucosa are not necessarily markers of tumor aggressiveness, but are useful for mapping tumor invasion, when it correlates to MRI and intraoperative observations by the surgeon. The proposed grading system also includes different grades according to non-invasive (grade 1; non-proliferative, 1a and proliferative, 1b) and invasive (grade 2; non-proliferative, 2a and proliferative, 2b) features and tumor spread (grade 3; cerebrospinal or systemic metastases). Obviously, correct assessment of tumor invasion is mandatory for this approach, as is evaluation of proliferation. Indeed, in our review of the literature we found a robust association between proliferation, assayed by Ki-67, and presence of invasiveness with 62% of revised reports suggesting a positive correlation [145,146,147] and 38% indicating an unclear association, albeit with a positive trend [13,148,149]. The controversial data on p53 and Ki-67 expression and correlation with invasiveness might in part be a result of differences in defining invasion, as reported by Sarkar et al. [140] and discussed herein. Extension and invasion of the lesion is also likely to be influenced by tumor size and anatomical characteristics, regardless of biological markers, at least in some cases. Indeed, as described, the proposed clinico-pathological grading system assigns invasive to grade 2a, but not to proliferative PitNETs. Thus, correlation between proliferation and invasiveness needs to be better defined and investigation on more sensitive and specific histological and/or biological markers associated with invasiveness are needed.

So far, the expression of several biological markers has been investigated by immunohistochemistry, with regards to their correlation with PitNET invasiveness and/or aggressiveness. However, to date no single marker has been found to be unequivocally related to tumor behavior. Liu et al. [150] recently described that EZH2 protein and mRNA expression are upregulated in invasive compared with non-invasive PitNETs, and that expression of EZH2 also correlated with the proliferation index. Interestingly, the same author also suggested that matrix metalloproteinase MMP-14 is significantly overexpressed in invasive PitNETs and correlates with EZH2 expression, indicating that both molecules are useful markers of invasiveness. In a recent report, MMP-14 has also been associated with the expression of ADAM12, described as a novel protein with a functional role for PitNET invasion [54]. The expression levels of other matrix metalloproteinases has been widely investigated. Upregulation of MMP-2, MMP-9, and ADAM-10 have been more frequently associated with tumor invasion [55,57,151,152,153]. Accordingly, metalloproteinase inhibitors (e.g., TIMP-3) are down-regulated in invasive PitNETs [154]. Growth factors are also reported to influence tumor growth and invasiveness. Fibroblast growth factors FGF2 and FGF4 are expressed in normal gland and regulate a large variety of functions, including cell differentiation, migration, and angiogenesis. In patients with sphenoid bone invasion, the relative expression of FGF2 was higher compared to patients without invasion [155]. On the other hand, FGF2 has been also reported to be downregulated in invasive PitNETs [156]. Tumors with loss of FGFR2 expression and presence of a truncated isoform of FGFR4, the pituitary tumor-derived FGFR4 (pdt-FGFR4), confer invasive growth of pituitary tumor cells and contributes to downregulation of N-cadherin expression [157,158]. Interestingly, loss of cytoplasmic expression of E-cadherin with translocation and accumulation to the nucleus has been reported to be a sign of aggressive behavior [159,160]. Angiogenesis and microvascular density have been also widely investigated. In a case of giant invasive prolactinoma refractory to treatment with dopamine agonists, immunohistological analysis revealed a strong immunoreactivity for both FGF-2 and VEGF, two potent angiogenic factors, and for the endothelial marker CD31, in line with other reports that suggest a higher degree of vascularization and high microvascular density (MVD) in invasive PitNETs [161,162]. Several other studies showed a relationship between angiogenic factors and tumor invasion and recurrence [163,164]. Nevertheless, so far antiangiogenic therapy for treatment of aggressive pituitary tumor has been of limited use [165]. Several gene expression and transcriptome studies have provided interesting data on pituitary cell lineage related factors and the pathogenesis of pituitary tumors [166,167,168,169]. Notwithstanding, the information obtained did not substantially contribute to better understanding of the mechanisms underlying the invasive behavior of PitNETs and did not reveal any useful markers for daily clinical practice. No specific association between invasiveness and hormone profile or lineage related transcription factors has been reported.

Recently, using gene microarray technology integrated with proteomics and transcriptomics datasets, Yu et al. [170] investigated the profile of differentially expressed genes between invasive and non-invasive non-functioning pituitary adenomas. These authors identified eight potential genes involved in invasion, including CAT, CLU, CHGA, EZR, KRT8, LIMA1, SH3GLB2, and SLC2A1 [170]. Cao et al. [171], using a computational bioinformatics analysis based on DNA microarray expression profiles reported a list of differentially expressed genes in invasive pituitary tumors that suggest two major class of molecules are involved with a significantly higher impact on invasion, namely the leukocyte transendothelial migration pathway and cell adhesion molecules. Specifically, they found six genes which appeared to be novel molecular biomarkers for invasion, which may be useful for diagnostic purposes: claudin 7 (CLDN7), contactin associated protein-like 2 (CNTNAP2), integrin, α6 (ITGA6), junctional adhesion molecule 3 (JAM3), protein tyrosine phosphatase, receptor type, C (PTPRC), and cadherin-associated protein α1 [171]. Recently, Joshi et al. [172] performed molecular screening of genes that are differentially expressed between invasive non-functioning adenoma samples and normal pituitary gland followed by pathway and ontology enrichment analyses. The data showed that invasive tumors preferentially upregulate genes releted to organ morphogenesis, extracellular matrix, and hormone activity, and downregulate those mainly associated with leukocyte chemotaxis, dendrites, and RAGE receptor binding. ESR1, SOX2, TTN, GFAP, WIF1, TTR, XIST, SPAG5, PPBP, AR, IL1R2, and HIST1H1C were found as the top hub genes in the upregulated and downregulated networks [172]. Immunohistochemical validation of the expression of putative biomarkers of invasion previously suggested by gene expression profiling studies is still limited. Using high-throughput tissue microarray analyses, Yao et al. [173] investigated the expression of nuclear receptor subfamily 2 group C member 2 (NR2C2), B cell translocation gene 2, T-box-19 (TBX19) and cyclin-dependent kinase 2 (CDK2) in both non-invasive and invasive non-functioning adenomas using immunohistochemistry. NR2C2 was found to be highly expressed in recurrent vs. primary tumors and was associated with invasion and progression, while TBX19 and CDK2 were only associated with recurrence [173].

MicroRNAs (miRNAs) have been also investigated with regards to tumor invasiveness. miRNA are small non-coding RNAs that may act as both tumor suppressors or oncogenes, depending on the type of cell type and target genes. Data from several reports has suggested that miRNA are deregulated in pituitary tumors and may play a role in invasiveness [174]. Liang et al. [175] identified a series of miRNAs (miR-329, miR-300, miR-381, miR-655) that target pituitary tumor transforming gene 1 (PTTG1) mRNA and inhibit protein expression. Evidence of a feedback loop between PTTG1 targeting miRNAs, PTTG1, and p53 that promotes pituitary tumorigenesis was also provided, but its significance with regards to invasiveness remains unclear [175]. In another recent report, Yu et al. suggested that miR-24, miR-93, miR-34a, and miR-126 are down-regulated in invasive pituitary adenomas compared with non-invasive lesions [176]. However, the role of miRNA in pituitary tumor invasiveness remains to be determined.

It is well known that primary central nervous system tumors contain tumor-initiating cells (TICs) with tumorigenic properties that contribute to maintainence of tumor growth and promote disease progression. A recent report investigated whether TICs also contribute to the growth of human pituitary tumors [177]. Using nanoString-based technology, the authors identified a differential stem cell gene expression profile within human pituitary adenomas of all hormonal subtypes that indicated CD15 as a putative pituitary adenoma-initiating cell marker. If up-regulation of putative stem cell markers, including CD15, is related to tumor invasiveness, this remains to be fully investigated. Finally, since familial cases of pituitary germline mutations responsible for the pathogenesis of PitNETs have been described, such as those in MEN1 or AIP, and such mutations may be present in a sporadic setting and correlate with tumor aggressiveness since tumors with MEN1 or AIP mutations have been reported to be resistant to conventional treatment [178]. Of note, a recent report indicated that MMP2 and MMP9 are upregulated in AIP-positive PitNETs and may confer a more aggressive phenotype [179]. In AIP-positive tumors, a significant number of altered mesenchymal-to-epithelial transition (EMT) associated genes, including epithelial markers (CDH1, CTNNB1, ERSP1, and EPCAM) and transcriptional (ZEB1) and post-transcriptional regulators (ESRP1) has been identified, suggesting that disruption of the EMT pathway may be responsible for a more aggressive phenotype. Moreover, in the same report, the authors provided experimental evidence highlighting the role of the microenvironment, and specifically of tumor-associated macrophages in promoting an EMT-like phenotype and enhancing cell migration and invasion in AIP-positive tumors.

## 3. Materials and Methods

**Figure 6:**
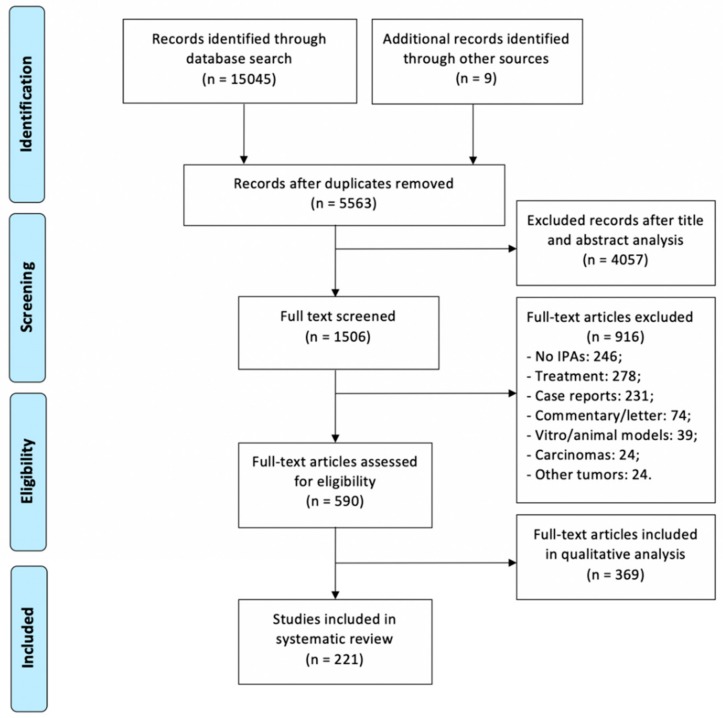
Literature review process, according to PRISMA [180].

Preferred reporting items for systematic reviews and meta-analyses (PRISMA) guidelines were followed (Figure 6). 

A systematic search of PubMed and Scopus electronic databases was conducted in July 2019 using the keywords: ‘pituitary adenoma’ and ‘pituitary’ in combination with ‘classification’, ‘cavernous sinus’, ‘invasion’ and ‘invasive*’. Only English studies published before July 2019 were included.

Papers describing pathological, molecular, radiological, and anatomical classifications and/or markers of PAs invasiveness. Exclusion criteria were: (1) pituitary adenomas without invasive features, 2) reports of therapies for invasive/aggressive PAs, (2) case reports, (3) letters to editor, (4) commentaries, (5) editorials.

Selected articles were subdivided in three groups: (1) those reporting radiological features of invasiveness, focusing on radiological classifications and their ability to predict invasion, (2) studies documenting the prognostic value of invasive behavior through molecular markers, (3) articles indicating anatomo-surgical considerations and their implication on tumor growth pathways.

## 4. Conclusions

PitNET invasiveness remains relatively understudied and there are several areas of controversy and research. The best grading system for PitNETs incorporates both pathological data and radiological classification of invasiveness [137], underlining that the study of invasiveness requires multidisciplinary evaluation that includes radiological investigations, intraoperative surgical study, and histology. This is particularly significant for growth of the tumor in the sphenoid sinus and cavernous sinus, as it is necessary to distinguish between extension of the tumor through a natural pathway and true invasiveness [7]. Further studies are needed to better define the histology and anatomy of the sellar and parasellar region, refine radiological diagnosis, and investigate the prognostic value of putative molecular markers of invasiveness.
